# No Association between Elevated Total Homocysteine Levels and Functional Outcome in Elderly Patients with Acute Cerebral Infarction

**DOI:** 10.3389/fnagi.2017.00070

**Published:** 2017-03-21

**Authors:** Wanjun Wang, Chunlin Gao, Changshen Yu, Shoufeng Liu, Dongzhe Hou, Yajing Wang, Chen Wang, Lidong Mo, Jialing Wu

**Affiliations:** ^1^Department of Neurorehabilitation, Department of Neurology, Tianjin Huanhu Hospital, Tianjin Key Laboratory of Cerebrovascular and Neurodegenerative DiseasesTianjin, China; ^2^Neurological Disease Biobank, Tianjin Neurosurgical Institute, Tianjin Huanhu Hospital, Tianjin Key Laboratory of Cerebrovascular and Neurodegenerative DiseasesTianjin, China

**Keywords:** homocysteine, cerebral infarction, functional outcome, elderly, predictor

## Abstract

**Background:** An elevated plasma total homocysteine (tHcy) level is an independent risk factor for vascular events. The aim of the present study was to investigate the association between tHcy levels in the acute phase of cerebral infarction and functional outcome among elderly patients.

**Methods:** Between October 2009 and December 2012, we recruited 594 elderly patients (age > 75) with first-onset acute cerebral infarction who were consecutively admitted to the Department of Neurology of Tianjin Huanhu Hospital, China. Levels of tHcy and other biochemical values were measured within 24 h after admission. tHcy values were classified according to quartiles (<9.94; 9.94 to <12.7; 12.7 to <16.8; and ≥16.8 μmol/L). We examined the relationship between tHcy levels at admission and modified Rankin Scale scores (mRS) using univariate and multivariate analyses. Patients were followed up at 3 months and 1 year after stroke.

**Results:** Within 3 months after stroke, 64 patients died, 37 had recurrent ischemic stroke, and 22 were lost to follow-up; thus, 471 patients were reviewed and analyzed. By the time of the 1-year follow-up, an additional 48 patients had died, 44 had recurrent ischemic stroke, and 40 had been lost to follow-up; the remaining 339 patients were thus reviewed and analyzed. Elevated tHcy levels were not associated with functional outcome among elderly patients with acute cerebral infarction (*p* > 0.05). Only the National Institutes of Health Stroke Scale score was associated with a poor outcome after adjusting for confounders at 3 months and 1 year (adjusted odds ratio, 1.38; 95% CI, 1.28–1.49; *p* < 0.01; adjusted odds ratio, 1.34; 95% CI, 1.25–1.44; *p* < 0.01, respectively).

**Conclusion:** Among elderly patients with acute cerebral infarction, elevated tHcy at admission was not a predictive factor of outcome at 3 months and 1 year after stroke onset.

## Introduction

Stroke is the second most common cause of death and the leading cause of disability worldwide, especially among aging patients (Lloyd-Jones et al., 2010). An estimated two million strokes occur each year in China, and the Chinese population is aging, with 200 million residents aged ≥65 years (Liu et al., 2011).

Some serum biomarkers, such as homocysteine (Hcy) have a predictive value in evaluating vascular events (Boushey et al., 1995; Eikelboom et al., 1999; Collaboration, 2002; Klerk et al., 2002; Wald et al., 2002). Hcy is a sulfhydryl-containing amino acid that is derived from the essential amino acid methionine, which is found in animal sources of protein (Hankey and Eikelboom, 1999).

A recent study shows that elevated tHcy levels could be associated with poor outcomes after stroke onset in women, but not in men (Zhong et al., 2016). However, little is known about the relationship between elevated tHcy and functional outcome among elderly patients with a cerebral infarction.

Therefore, the aim of the present study was to investigate the association between tHcy levels in the acute phase of cerebral infarction and functional outcome among elderly patients.

## Materials and Methods

The present study received the approval of the Ethics Committee of Tianjin Huanhu Hospital, and all procedures were in accordance with the ethical standards set forth by the committee. Informed consent was obtained from all individual participants included in the study. The present study is observational studies, without giving extra intervention on the patients.

### Patient Selection

We used a hospital-based registry of consecutive patients with first-onset acute cerebral infarction from October 2009 to December 2012. All patients were admitted into the Department of Neurology of Tianjin Huanhu Hospital, which is a specialized neurology hospital in Tianjin, China. Diagnosis of acute cerebral infarction was made according to the criteria of the World Health Organization (Schlegel et al., 2003). The inclusion criteria included a first ischemic stroke within 7 days, which was confirmed by imaging studies of the head (magnetic resonance imaging or computed tomography). Exclusion criteria for the present study were an age ≤75 years, B vitamin and/or folic acid therapy within 2 weeks of hospital admission, renal failure, or an unwillingness to participate.

Detailed baseline demographics and clinical characteristics were collected and recorded during the patient hospitalization. Patients or their authorized proxies were followed up at 3 months and 1 year after stroke onset through either telephone or face-to-face interviews. Follow-up results were immediately recorded into the stroke database.

**FIGURE 1:**
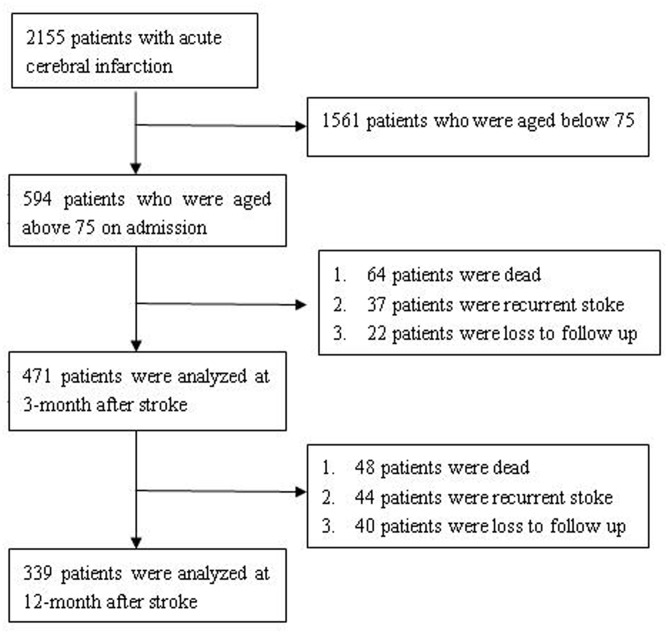
**How chart of patient selection**.

We retrospectively reviewed and extracted data from the registry. Among 2,155 patients with acute cerebral infarction who were admitted to the hospital within 7 days of stroke onset, 1,561 patients who were aged ≤75 years were excluded from the analysis. A total of 594 patients aged >75 years with acute cerebral infarction were included in the study. Finally, 471 patients were followed up at 3 months, and 339 patients were followed up at 1 year (**Figure 1**).

### Demographic and Clinical Characteristics

Baseline information and stroke risk factors for all patients were collected within 24 h of admission. The baseline information included age, sex, height, and weight data. Stroke risk factors included a clinical medical history of hypertension, diabetes mellitus, atrial fibrillation, dyslipidemia, or cerebral artery stenosis. We also assessed patients’ modifiable lifestyle factors, including current smoking status (≥1 cigarette per day for 1 year) and current alcohol drinker status (≥1 drink per week for 1 year). Body mass index was measured according to body weight and height, and obesity was defined as a body mass index ≥30 kg/m^2^.

The stroke subtypes were classified according to the Trial of Org 10172 in Acute Stroke Treatment (TOAST) criteria, and were defined as atherothrombotic, cardioembolic, lacunar, other causes, and undetermined (Adams et al., 1993). The National Institutes of Health Stroke Scale (NIHSS) was evaluated at the time of admission. The modified Rankin Scale (mRS) was evaluated at 3 months and 1 year after stroke onset.

### Laboratory Methods

Blood biochemical variables were measured following a fast of at least 8 h on the first day after admission. Levels of tHcy were measured using nephelometric technology, which was conducted on a BNII system (SIEMENS, Erlangen, Germany). Serum high-sensitivity C-reaction protein (hsCRP) concentration was measured using an immunoturbidimetric assay. Fasting glucose was determined using the glucose oxidase method. All variables were analyzed in a certified central laboratory.

Quartile classification is the most common statistical layering method; therefore tHcy values were classified into four groups according to quartiles (<9.94; 9.94 to <12.7; 12.7 to <16.8; and ≥16.8 μmol/L).

### Study Outcome

To evaluate functional outcome in elderly patients, functional impairment was graded using the mRS. A favorable outcome was defined as an mRS score of 0–2 at 3 months and 1 year after stroke onset. A poor outcome was defined as an mRS score of 3–5, which indicates that patients cannot live independently.

### Statistical Analysis

In the present study, categorical variables are reported as counts and percentages, and continuous variables are reported as median values or means ± standard deviations. Normality of distribution was assessed with frequency histograms and normal probability plots. The univariate associations between variables of interest were evaluated using a Student’s *t*-test, Kruskal–Wallis test, and chi-square test, as appropriate. Covariate-adjusted associations between the variables of interest were assessed using a logistic regression analyses. The selection of the potentially confounding covariates was based on the existing knowledge about their potential relationship with the variable in focus. Two-tailed tests of significance were performed, and *p*-values < 0.05 were considered statistically significant. The software package SPSS version 21.0 was used to perform statistical analyses.

## Results

### Baseline Demographics and Clinical Characteristics According to Quartiles of tHcy Levels

Baseline demographics and clinical characteristics according to quartiles of tHcy levels are listed in **Table 1**. A total of 594 elderly patients (323 men [54.4%]; median age, 78 years) were included in the present study. The mean tHcy level of all elderly patients was 15.61 ± 10.77 mmol/L (range, 2.42–105.00 μmol/L); a total of 437 (73.6%) patients had hypertension, 172 (29.0%) had diabetes mellitus, 91 (15.3%) had atrial fibrillation, 125 (21.0%) had dyslipidemia, and 130 (21.9%) had cerebral arterial stenosis. Regarding the modifiable lifestyle factors, 123 (20.7%) patients were current smokers, 36 (6.1%) were alcohol drinkers, and 101 (17.0%) were obese. Elderly patients with elevated tHcy levels were more likely to be male than patients with lower tHcy levels. Stroke subtypes were equally distributed among the Hcy quartiles. There was no statistically significant difference among the stratifications with regard to stroke severity and the mean time from stroke onset (*p* = 0.693, *p* = 0.888, respectively).

**Table 1:** Baseline characteristics of all patients according to quartiles of tHcy levels.

	Q1 (<9.94), *n* = 150	Q2 (9.94–12.7), *n* = 149	Q3 (12.7–16.8), *n* = 147	Q4 (>16.8), *n* = 148	*P*
Age, year^∗^	78	78	79	79	0.768
Male, *n* (%)	68 (45.3)	68 (45.3)	91 (61.9)	96 (64.9)	0.000
Hypertension, *n* (%)	111 (74.0)	106 (71.1)	102 (69.4)	118 (79.7)	0.197
DM, *n* (%)	49 (32.7)	53 (35.6)	36 (24.5)	34 (23.0)	0.043
AF, *n* (%)	30 (20.0)	22 (14.8)	23 (15.6)	16 (10.8)	0.179
Dyslipidemia, *n* (%)	42 (28.0)	28 (18.8)	25 (17.0)	30 (20.3)	0.097
CAS, *n* (%)	32 (21.3)	23 (15.4)	35 (26.9)	40 (27.0)	0.100
Smoking, *n* (%)	27 (18.0)	28 (18.8)	28 (19.0)	40 (27.0)	0.183
Alcohol drinker, *n* (%)	7 (4.7)	7 (4.7)	10 (6.8)	12 (8.1)	0.526
Obesity, *n* (%)	27 (18.0)	28 (18.8)	21 (14.3)	25 (16.9)	0.750
TOAST subtype					0.859
LA	73 (48.7)	82 (55.0)	77 (52.4)	85 (57.4)	
CE	10 (6.7)	15 (10.1)	15 (10.2)	14 (9.5)	
SA	47 (31.3)	32 (21.5)	36 (24.5)	32 (21.6)	
OE	10 (6.7)	9 (6.0)	9 (6.1)	7 (4.7)	
UE	10 (6.7)	11 (7.4)	10 (6.8)	10 (6.9)	
CHO, mmol/L^∗^	4.79 (1.29)	4.84 (1.53)	4.37 (1.32)	4.71 (1.21)	0.147
TG, mmol/L^∗^	1.06 (0.62)	1.05 (0.75)	1.03 (0.77)	1.07 (0.75)	0.983
HDL-C, mmol/L^∗^	1.01 (0.35)	1.04 (0.42)	1.02 (0.31)	0.98 (0.32)	0.599
LDL-C, mmol/L^∗^	3.17 (1.20)	2.95 (1.12)	2.73 (1.05)	3.03 (1.08)	0.268
FG, mmol/L^∗^	5.83 (2.93)	5.61 (2.30)	5.50 (2.18)	5.57 (1.53)	0.166
hsCRP, mg/L^∗^	4.34 (15.72)	4.26 (8.31)	3.97 (12.25)	5.29 (15.36)	0.357
Hcy, μmol/L	8.75 (1.58)	11.30 (1.25)	14.50 (2.00)	22.40 (14.35)	<0.001
NIHSS^∗^	6.50 (10.75)	6.00 (9.00)	7.00 (11.00)	8.00 (9.00)	0.693
Time ^#^	83.04 ± 50.94	80.38 ± 53.59	80.97 ± 49.87	84.65 ± 52.70	0.888


*DM, diabetes mellitus; AF, atrial fibrillation; CAS, cerebral artery stenosis; CHO, cholesterol; TG, triglycerides; HDL-C, high-density lipoprotein cholesterol; LDL-C, low-density lipoprotein cholesterol; FG, fasting glucose; hsCRP, high sensitivity C-reactive protein; Hcy, homocysteine. NIHSS, National Institutes of Health Stroke Scale.*

*Time represents hours from stroke onset.*

*^∗^Median (interquartile range); ^#^Mean ± standard deviation.*

### Results at a 3-Month Follow-up

A total of 64 (10.8%) patients died within 3 months of stroke onset. Among these patients, 17 patients (16 from cerebral infarction and complications, 1 from acute myocardial infarction) died during hospitalization, and 47 patients (36 from cardio-cerebral vascular events and complications, 11 from other causes) died during the first 3 months of follow-up. Thirty-seven patients had recurrent ischemic stroke, and 22 patients were lost to follow-up (**Figure 1**). At the 3-month follow-up, 471 patients responded by telephone or in-person interview and were included in the analysis. There were 246 patients with mRS scores of 0–2 and 225 patients with mRS scores of 3–5. Univariate analyses demonstrated that total cholesterol, low density lipoprotein cholesterol, hsCRP, fasting blood glucose, and NIHSS score were associated with a poor outcome; however, only NIHSS score was associated with a poor outcome after adjusting for confounders (adjusted odds ratio [OR], 1.38; 95% CI, 1.28–1.49; *p* < 0.01). Total cholesterol, low-density lipoprotein cholesterol, hsCRP, time from stroke onset, and fasting blood glucose levels had no relationship with a poor outcome after adjusting for confounders. There was no significant difference in outcome among patients grouped by tHcy quartile at a 3-month follow-up (**Table 2**).

**Table 2:** Predictors of poor functional outcome in elderly patients with acute cerebral infarction.

3 months	Unadjusted OR (95% CI)	*P*	Adjusted OR (95% CI)	*P*
CHO, mmol/L	1.25 (1.05–1.48)	0.01	0.81 (0.39–1.70)	0.58
LDL-C, mmol/L	1.37 (1.10–1.69)	<0.01	1.74 (0.73–4.15)	0.70
hsCRP, mg/L	1.01 (1.00–1.02)	0.01	0.99 (0.98–1.01)	0.22
FG, mg/L	1.20 (1.08–1.35)	<0.01	1.09 (0.94–1.27)	0.26
NIHSS	1.35 (1.27–1.43)	<0.01	1.38 (1.28–1.48)	<0.01^∗^
Time	1.00 (0.99–1.01)	0.26	0.99 (0.99–1.01)	0.78
**tHcy^∗^**				
Q1	1		1	
Q2	1.50 (0.90–2.50)	0.12	0.50 (0.17–1.50)	0.21
Q3	0.93 (0.55–1.56)	0.78	0.77 (0.28–2.10)	0.61
Q4	1.55 (0.77–2.15)	0.33	0.73 (0.27–1.94)	0.52
**1 year**				
AF, (%)	2.04 (1.08–3.87)	0.03	0.96 (0.39–2.33)	0.92
LDL-C, mmol/L	1.26 (0.98–1.62)	0.08	1.26 (0.93–1.72)	0.14
NIHSS	1.34 (1.25–1.43)	<0.01	1.34 (1.25–1.43)	<0.01^#^
Time	1.00 (1.00–1.01)	0.068	1.00 (0.99–1.01)	0.28
**tHcy^#^**				
Q1	1		1	
Q2	1.05 (0.56–1.97)	0.87	1.16 (0.51–2.63)	0.73
Q3	0.85 (0.45–1.62)	0.63	1.25 (0.56–2.79)	0.59
Q4	1.07 (0.57–2.01)	0.83	0.98 (0.43–2.21)	0.96


*CI indicates confidence interval; OR, odd ratio. Time represents hours from stroke onset.*

^∗^*Adjusted for total cholesterol, low-density lipoprotein cholesterol level, high-sensitivity C-reactive protein level, fasting glucose, NIHSS and time.*

*^#^Adjusted for atrial fibrillation, low-density lipoprotein cholesterol, NIHSS and time.*

### Results at a 1-Year Follow-Up

Among the 471 patients available for follow-up at 3 months, 48 (10.2%) patients died, 44 (9.3%) patients had recurrent ischemic stroke, and 40 (8.5%) patients were lost at a 1-year follow-up at 1 year. Thirty-two patients died from cardio-cerebral vascular events and complications, and 16 died from other causes (**Figure 1**). A total of 339 patients responded by telephone or in-person interview at 1 year and were included in the analysis. There were 220 patients with mRS scores of 0–2, and 119 patients with mRS scores of 3–5. Univariate analyses demonstrated that atrial fibrillation, low-density lipoprotein cholesterol levels, and NIHSS score were associated with a poor outcome; however, only NIHSS score was associated with a poor outcome after adjusting for confounders (adjusted odds ratio, 1.34; 95% CI, 1.25–1.44; *p* < 0.01). Atrial fibrillation, time from stroke onset, and low-density lipoprotein cholesterol levels had no relationship with a poor outcome after adjusting for confounding variables. There was no significant difference in outcome among patients grouped by tHcy quartile at a 1-year follow-up (**Table 2**).

## Discussion

Some neurological scales, such as the NIHSS and mRS (Schlegel et al., 2003; Wilson et al., 2005; Bruno et al., 2013; Kim et al., 2013), can predict stroke patient outcomes. However, it is very difficult to evaluate patient outcomes using scales when patients suffer from apraxia, aphasia, or disorientation. In addition, specific serum biomarkers also have predictive value in evaluating stroke outcome (Yang et al., 2014; Yoo et al., 2014). Therefore, it is important for clinicians to evaluate functional impairment and predict patient outcome after ischemic stroke, as this would help in determining early-stage treatment and rehabilitation methods.

Stroke severity is a well-known predictor of stroke outcome (Veerbeek et al., 2011). Furthermore, the clinical predictive validity of the NIHSS score has been shown in several investigations (Schlegel et al., 2003; Kwah et al., 2013). Consistent with other studies (Adams et al., 1999; Kwah et al., 2013), we also found that initial NIHSS score was an independent predictor (OR, 1.38; *p* < 0.01) of patient outcome at 3 months and (OR, 1.34; *p* < 0.01) 1 year after stroke.

A previous study reported that serum Hcy is an independent risk factor for stroke (Pang et al., 2014). The mechanisms explaining the relationship between hyperhomocysteinemia and stroke are not yet fully understood. Hyperhomocysteinemia could induce changes in mRNA and protein expression of CRP in vascular smooth muscle cells via the NMDAr-ROS-ERK1/2/p38-NF-κB signal pathway (Kim et al., 2013). Moreover, an increased serum Hcy level is an early atherosclerotic promoter. Hyperhomocysteinemia causes cardiovascular disease through not only the proliferation of vascular smooth muscle cells but also the acceleration of endothelial dysfunction, platelet coagulation, and cholesterol synthesis (Stuhlinger et al., 2001; Ke et al., 2010; Shenoy et al., 2014). The findings on the relationship between hyperhomocysteinemia and outcome after stroke have been inconsistent (Del Ser et al., 2001; Faeh et al., 2006). Moreover, trials investigating the effect of B vitamins, which reduce tHcy levels, on endothelial function and outcome of patients with cardiovascular disease have shown conflicting results (Chambers et al., 2000; Title et al., 2000; Woo et al., 2002; van Dijk et al., 2016). The heterogeneous methodologies included in these studies may partially explain discrepancies in the final results. For example, differences in time of evaluation, age and sex of recruited patients, the localization of the stroke area, outcome measures, and type of stroke may result in different results.

There is debate about whether tHcy is a causative risk factor in stroke or is merely a secondary marker of risk in survivors. An evaluation of tHcy both before and after acute stroke would help to answer this question. Given the difficulty in predicting the onset of stroke, however, these data are unavailable. The measurement of tHcy concentrations immediately after acute stroke is necessary because an observation of elevated tHcy levels at this time would be more suggestive of a causal association than the occurrence of hyperhomocysteinemia in survivors sampled at convalescent phases after stroke. Some studies report that tHcy concentrations are not elevated after acute stroke, but rise significantly at convalescent phases (Lindgren et al., 1995; Meiklejohn et al., 2001). Our findings are in accordance with the Lindgren et al. (1995) study; both studies recruited elderly patients (median age, 78 vs.75 years) at the acute phase (mean of 3 vs. 2 days after stroke onset), and tHcy concentration was comparable (mean of 15.61 vs.13.4 μmol/L).

In the present study, both univariate and multivariate logistic regression analyses indicated that an elevated Hcy level at admission was not a predictive factor among elderly patients with acute cerebral infarction. Similar findings reported that plasma Hcy levels have no value as predictors of functional disability in Asian patients with stroke (Mizrahi et al., 2005; Song et al., 2009). However, the Mizrahi et al. (2005) study assessed patient outcome with Function Independent Measure scores instead of mRS scores, and those two studies recruited both younger and elderly patients. One possible explanation for this finding is that the association between Hcy level and stroke is not causal among elderly patients. Alternatively, the harmful effects of hyperhomocysteinemia could be masked by other vascular risk factors, such as hypertension and diabetes. The patients included in the present study were elderly, therefore the prevalence rates of hypertension and diabetes were 74.9 and 29.5%, respectively, which were much higher than rates than those reported in other studies. However, there may be a causal link between hyperhomocysteinemia and hypertension because elevated Hcy levels may lead to hypertension through the induction of a diastolic dysfunction of vessels and a reduction of vascular wall flexibility. In addition, we failed to find any relationship between the Hcy level and serum hsCRP level, indicating that elevated Hcy does not lead to additional inflammatory responses among elderly patients.

Some studies report that elevated Hcy levels can cause a more severe stroke via multiple mechanisms, such as platelet activation, inflammation, prothrombotic disorders, and fibrinolysis (Stamler et al., 1993; Parnetti et al., 2004; Tay et al., 2006). However, in accordance with the study by Perini et al. (2005), we found no association between plasma tHcy and stroke severity, suggesting that elevated tHcy is not associated with stroke severity in our cohort of patients.

Another finding of the present study was that the percentage of men was much higher in the two highest tHcy quartiles than in the two lowest quartiles (65.7% vs. 44.5%). This result could be explained by the fact that estrogen or estrogen associated with progestin may have a positive role in decreasing cardiovascular risk due to a significant reduction in Hcy levels (Lakryc et al., 2015).

There are a few limitations of the current study, as it was retrospective and used data obtained from a single hospital. Moreover, the present study was performed in hyperhomocysteinemic elderly patients; therefore, our results may not be representative of the general population. This design may have resulted in an inevitable selection bias. Regretfully, another limitation is the lack of data on repeated measurements of Hcy levels during the follow-up period. Future studies with larger patient populations are necessary to assess the prognostic value of tHcy levels after acute cerebral infarction.

## Conclusion

In summary, in the present study, we observed that an elevated tHcy level at admission was not a predictive factor of the outcome at 3 months and 1 year after acute cerebral infarction among elderly patients. These results indicate that it is necessary to treat acute cerebral infarction on an individual basis in elderly patients, especially when therapies to normalize plasma tHcy levels are considered.

## Author Contributions

JW contributed to the conception and design of the work; WW, CG, CY, SL, DH, YW, CW, and LM contributed the data acquisition; JW and WW contributed the analysis and interpretation of data for the work; WW and CG contributed drafting the work, JW contributed revising the work for important intellectual content. All authors approved of the final version to be published, and agree to be accountable for all aspects of the work in ensuring that questions related to the accuracy or integrity of any part of the work are appropriately investigated and resolved.

## Conflict of Interest Statement

The authors declare that the research was conducted in the absence of any commercial or financial relationships that could be construed as a potential conflict of interest.
